# Comparative Assessment of Various Low-Dissipation Combined Models for Three-Terminal Heat Pump Systems

**DOI:** 10.3390/e23050513

**Published:** 2021-04-23

**Authors:** Zhexu Li, Haibo Cao, Hanxin Yang, Juncheng Guo

**Affiliations:** College of Physics and Information Engineering, Fuzhou University, Fuzhou 350116, China; n191120059@fzu.edu.cn (Z.L.); 201120016@fzu.edu.cn (H.C.); t11062@fzu.edu.cn (H.Y.)

**Keywords:** multi-terminal devices, comparative assessment, low-dissipation assumption, upper and lower bound, optimal construction

## Abstract

Thermally driven heat pump systems play important roles in the utilization of low-grade thermal energy. In order to evaluate and compare the performances of three different constructions of thermally driven heat pump and heat transformer, the low-dissipation assumption has been adopted to establish the irreversible thermodynamic models of them in the present paper. By means of the proposed models, the heating loads, the coefficients of performance (COPs) and the optimal relations between them for various constructions are derived and discussed. The performances of different constructions are numerically assessed. More importantly, according to the results obtained, the upper and lower bounds of the COP at maximum heating load for different constructions are generated and compared by the introduction of a parameter measuring the deviation from the reversible limit of the system. Accordingly, the optimal constructions for the low-dissipation three-terminal heat pump and heat transformer are determined within the frame of low-dissipation assumption, respectively. The optimal constructions in accord with previous research and engineering practices for various three-terminal devices are obtained, which confirms the compatibility between the low-dissipation model and endoreversible model and highlights the validity of the application of low-dissipation model for multi-terminal thermodynamic devices. The proposed models and the significant results obtained enrich the theoretical thermodynamic model of thermally driven heat pump systems and may provide some useful guidelines for the design and operation of realistic thermally driven heat pump systems.

## 1. Introduction

The generalized models and the associated performance boundaries of thermodynamic devices are desired for researchers. Within the frame of quasi-static assumption, a series of classical thermodynamic models have been proposed. Based on these models, the upper bounds of efficiency and coefficient of performance (COP) for heat engine, refrigerator and heat pump are obtained, i.e., the Carnot efficiency and Carnot COP, which are deemed as the cornerstone of thermodynamics. However, the quasi-static processes imply the time duration for completing a full cycle is infinite, which indicates the value of the Carnot efficiency and COP in practice is limited [1].

To achieve finite power output, cooling power and heating load, endoreversible assumption has been adopted to construct finite-time cycles [2,3,4,5]. With the help of the endoreversible Carnot heat engine model, the famous CA efficiency (efficiency at maximum power output) has been derived [3]. Unfortunately, it has been proved in the following research that the performances of the endoreversible thermodynamic models are closely dependent on the law of heat transfer [6]. In other words, the CA efficiency is not universal. The drawback of the endoreversible thermodynamic model motivates researchers to keep exploring the way of establishing a more universal thermodynamic model beyond specific heat transfer mechanisms.

Recently, an original low-dissipation thermodynamic cycle model was proposed by Esposito et al. [7] by noticing the first order time-related entropy generation of many irreversible thermodynamic processes behaves as 1/t [7,8]. In this model, instead of specifying the heat-transfer law, two parameters including the information of irreversibility are introduced. More importantly, at two extreme asymmetry conditions, the upper and lower bounds of the efficiency at maximum power output fitting well with practical cases are first obtained [7,9,10]. The generality and practicability of low-dissipation model led to the thriving research. It has been further adopted to discuss the performances of various thermodynamic devices, such as Carnot-like refrigerator [11], Carnot-like engine [12], quantum heat engine [13,14] and chemical engines [15]. In addition, various constraints [16,17,18] have been considered in the performance discussions of the low-dissipation thermal devices. In addition, different objective functions [19,20,21] have been introduced to provide more comprehensive optimization criterions. Furthermore, the relations between low-dissipation model, minimally nonlinear irreversible thermodynamic model [22,23] and endoreversible model [24,25,26] have attracted the attentions of researchers.

Note that all the applications of low-dissipation assumption in the previous research focus on the thermodynamic devices operating between only two reservoirs. However, multi-terminal thermodynamic systems play the important roles in the utilization of low-grade energies [27,28,29,30] and the energy resources at microscopic scale [31,32]. In this regard, the low-dissipation assumption has been used to construct the combined cycle models of thermally driven refrigerator and heat pump by considering the constraint of reversible entropies inside two subsystems in the last two years [33,34], which obtains the bounds of the COP at maximum cooling power and heating load for the first time and extends the application scope of low-dissipation model to multi-terminal thermodynamic systems.

**Figure 1:**
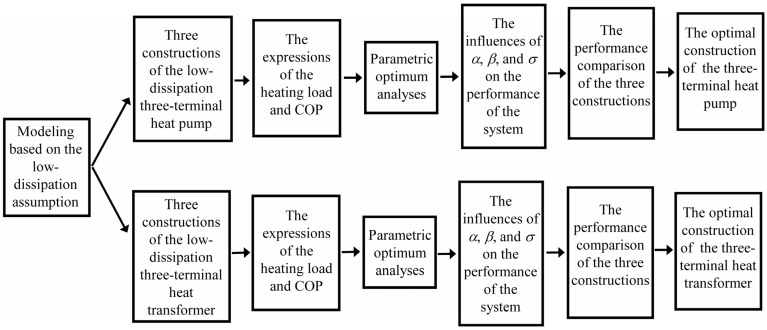
The comparative schematic of the organization and the research strategy of the present paper.

It is noteworthy to point out that there are three equivalent combined constructions for the three-terminal thermodynamic devices under reversible limit [35]. With the help of endoreversible assumption, the performances of different constructions of the three-terminal heat pump, heat transformer, chemical pump and chemical potential transformer are investigated, respectively. In addition, the optimal constructions for these three-terminal thermodynamic devices have been determined, respectively [36,37,38]. Attending the above comments, it is natural to ask: How to evaluate and compare the performances of the low-dissipation three-terminal thermodynamic devices with different combined constructions; whether the optimal combined constructions of those three-terminal thermodynamic devices based on low-dissipation assumption are consistent with Refs. [36,37,38]; whether the optimal combined constructions in accordance with practical thermally driven heat pump [39,40,41] and thermal driven heat transformer can be deduced [42,43] within the frame of low-dissipation assumption? To find out the answers to the above questions will further reveal the compatibility between the low-dissipation model and endoreversible model for multi-terminal thermodynamic devices and clarify the validity of the application of low-dissipation model for multi-terminal thermodynamic devices, which is the main objective of the present paper. In order to present the organization and the research strategy of the present paper from an overall point of view, a comparative schematic figure is given in Figure 1.

In the present paper, three combined constructions of the three-terminal heat pump and heat transformer are first presented with the help of the low-dissipation assumption, respectively. Based on the proposed low-dissipation models, the heating loads and the COPs for various constructions are derived, followed by the discussions of the optimal relations between them. The performances of different constructions of the systems are numerically assessed and the optimal operation regions and the influences of several parameters on the performances of the systems are determined and investigated. More importantly, according to the results obtained above, the upper and lower bounds of the COP at maximum heating load for different constructions of the systems are generated and compared. Accordingly, the optimal constructions for the three-terminal heat pump and heat transformer are determined, which are accordant with Refs. [36,37,38] and practical thermally driven heat pump systems. The results confirm the consistency between the low-dissipation model and endoreversible model and highlights the validity of the application of low-dissipation model for multi-terminal thermodynamic devices. In the end, the important conclusions are summarized.

## 2. Model Descriptions

**Figure 2:**
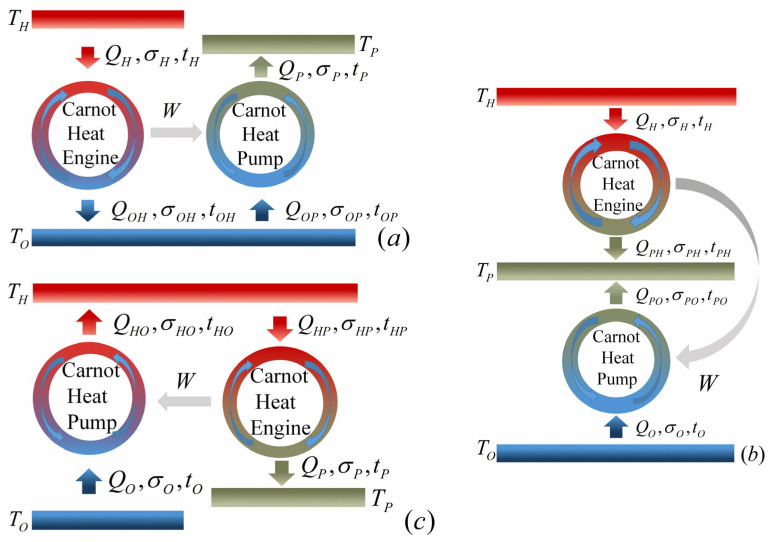
The low-dissipation models of three combined constructions for three-terminal heat pump. (**a**–**c**) represent models *A*, *B* and *C*, respectively.

**Figure 3:**
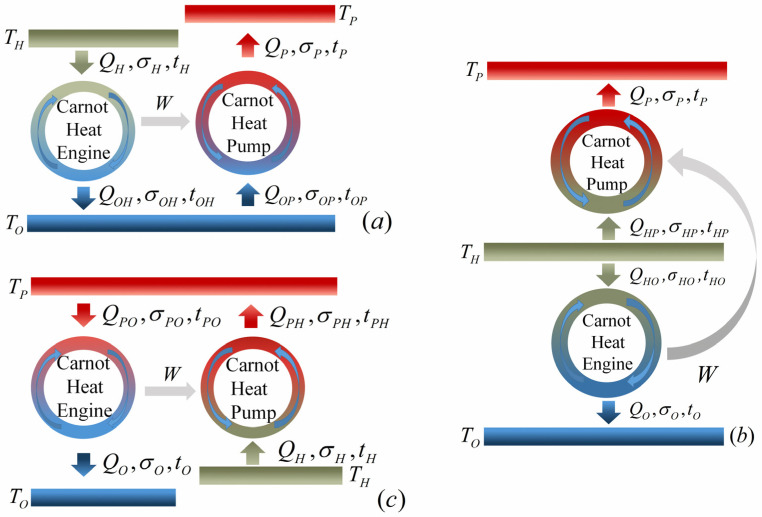
The low-dissipation models of three combined constructions for three-terminal heat transformer. (**a**–**c**) represent models *A*, *B* and *C*, respectively.

For the heat pump system operating between three terminals, namely, high-temperature source, heated space and environment, with temperatures $T_{H}$, $T_{P}$ and $T_{O}$, respectively, there are two configurations determining by the values of $T_{H}$ and $T_{P}$. To be specific, when $T_{H}>T_{P}$, more heat with lower grade is pumped into the heated space comparing to the heat absorbed from the high-temperature source. In contrast, when $T_{H}<T_{P}$, less heat with higher grade is obtained by the heated space in comparison with the heat released by the high-temperature source. These two aforementioned configurations are usually named as heat pump and heat transformer, respectively. Figure 2a–c show three possible combined constructions for a three-terminal heat pump. It has been proven that these three constructions are equivalent under reversible limit [35]. Similarly, there are three possible constructions for a three-terminal heat transformer shown by Figure 3a–c which are equivalent under reversible limit as well [35]. In addition, the COP for both three-terminal heat pump and heat transfer under reversible limit is given by [35]

(1) $$\psi_{r}=\frac{Q_{P}}{Q_{H}}=\frac{T_{P}}{T_{H}}\frac{T_{H}-T_{O}}{T_{P}-T_{O}}.$$

Nevertheless, the heating load vanishes for reversible three-terminal heat pump and heat transformer [35]. Consequently, the low-dissipation assumption will be used to establish more practical models of three-terminal heat pump and heat transformer and explore the performance characteristics and differences of various constructions in the following.

Before establishing the low-dissipation models of three-terminal heat pump and heat transformer, it is necessary to briefly describe the working processes of the absorption heat pump, as an example of the thermally driven heat pump systems, for better understanding of the systems.

**Figure 4:**
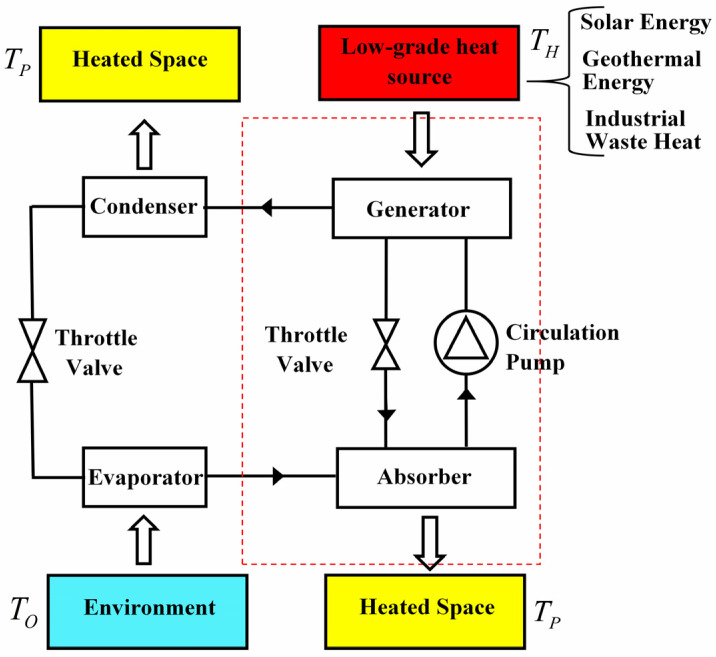
The schematic diagram of the absorption heat pump.

**Figure 5:**
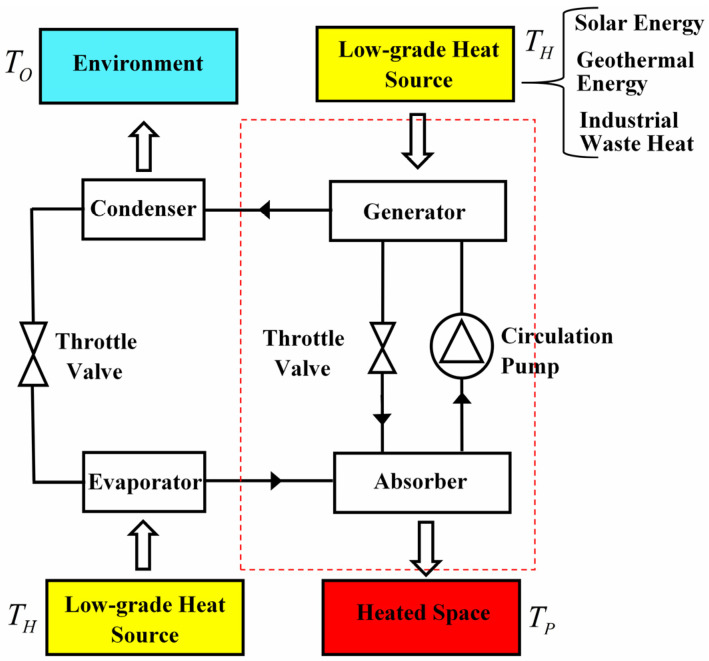
The schematic diagram of the absorption heat transformer.

Figure 4 shows the schematic diagram of an absorption heat pump operating between three heat sources. Low-grade thermal energy is used to drive this system rather than electricity. Specifically, heat is absorbed by the refrigerant-absorbent mixture from low-grade heat source in order to separate the refrigerant in the generator. And then, the refrigerant is transported to the condenser and releases heat into heated space. After that the refrigerant absorbs heat from environment in the evaporator. In the end, the refrigerant transported from evaporator is absorbed by the absorbent in the absorber and releases heat into the heated space. After going through a full cycle, heats released by the high-temperature reservoir and absorbed from environment are both pumped into the heated space without consuming electricity. The working processes of the absorption heat transformer are similar except the heat reservoirs contacting with generator, condenser, evaporator and absorber are changed, which can be seen from Figure 5.

### 2.1. Low-Dissipation Three-Terminal Heat Pump

Based on the low-dissipation assumption, when the three-terminal heat pump is constructed as models *A*, *B* and *C* (shown by Figure 2a–c, respectively) the heats exchanged between the system and the three heat reservoirs in four heat transferring processes can be expressed as [7]

(2) $$\left\{ \begin{array}{l} Q_{H}^{a}=Q_{Hr}^{a}(1-\frac{\sigma_{H}^{a}}{t_{H}^{a}})=T_{H}\Delta S_{he}^{a}(1-\frac{\sigma_{H}^{a}}{t_{H}^{a}})\\ Q_{OH}^{a}=Q_{OHr}^{a}(1+\frac{\sigma_{OH}^{a}}{t_{OH}^{a}})=T_{H}\Delta S_{he}^{a}(1+\frac{\sigma_{OH}^{a}}{t_{OH}^{a}})\\ Q_{P}^{a}=Q_{Pr}^{a}(1+\frac{\sigma_{P}^{a}}{t_{P}^{a}})=T_{P}\Delta S_{hp}^{a}(1+\frac{\sigma_{P}^{a}}{t_{P}^{a}})\\ Q_{OP}^{a}=Q_{OPr}^{a}(1-\frac{\sigma_{OP}^{a}}{t_{OP}^{a}})=T_{O}\Delta S_{hp}^{a}(1-\frac{\sigma_{OP}^{a}}{t_{OP}^{a}}) \end{array}\right.,$$

(3) $$\left\{ \begin{array}{l} Q_{H}^{b}=Q_{Hr}^{b}(1-\frac{\sigma_{H}^{b}}{t_{H}^{b}})=T_{H}\Delta S_{he}^{b}(1-\frac{\sigma_{H}^{b}}{t_{H}^{b}})\\ Q_{PH}^{b}=Q_{PHr}^{b}(1+\frac{\sigma_{PH}^{b}}{t_{PH}^{b}})=T_{P}\Delta S_{he}^{b}(1+\frac{\sigma_{PH}^{b}}{t_{PH}^{b}})\\ Q_{PO}^{b}=Q_{POr}^{b}(1+\frac{\sigma_{PO}^{b}}{t_{PO}^{b}})=T_{P}\Delta S_{hp}^{b}(1+\frac{\sigma_{PO}^{b}}{t_{PO}^{b}})\\ Q_{O}^{b}=Q_{Or}^{b}(1-\frac{\sigma_{O}^{b}}{t_{O}^{b}})=T_{O}\Delta S_{hp}^{b}(1-\frac{\sigma_{O}^{b}}{t_{O}^{b}}) \end{array}\right.,$$

and

(4) $$\left\{ \begin{array}{l} Q_{HP}^{c}=Q_{HPr}^{c}(1-\frac{\sigma_{HP}^{c}}{t_{HP}^{c}})=T_{H}\Delta S_{he}^{c}(1-\frac{\sigma_{HP}^{c}}{t_{HP}^{c}})\\ Q_{HO}^{c}=Q_{HOr}^{c}(1+\frac{\sigma_{HO}^{c}}{t_{HO}^{c}})=T_{H}\Delta S_{hp}^{c}(1+\frac{\sigma_{HO}^{c}}{t_{HO}^{c}})\\ Q_{P}^{c}=Q_{Pr}^{c}(1+\frac{\sigma_{P}^{c}}{t_{P}^{c}})=T_{P}\Delta S_{he}^{c}(1+\frac{\sigma_{P}^{c}}{t_{P}^{c}})\\ Q_{O}^{c}=Q_{Or}^{c}(1-\frac{\sigma_{O}^{c}}{t_{O}^{c}})=T_{O}\Delta S_{hp}^{c}(1-\frac{\sigma_{O}^{c}}{t_{O}^{c}}) \end{array}\right.,$$

respectively. In Equations (2)–(4), $Q_{ir}^{k}$ (i=H,OH,P,OP for k=a; i=H,PH,PO,O for k=b; and i=HP,HO,P,O for k=c) are the exchanged heats in the four heat transferring processes under reversible limit, $\Delta S_{j}^{k}$ (*j* = *he*, *ph* and k=a,b,c) are the corresponding reversible entropy changes, $t_{i}^{k}$ (i=H,OH,P,OP for k=a; i=H,PH,PO,O for k=b; and i=HP,HO,P,O for k=c) are the time durations of four heat exchanging processes and $\sigma_{i}^{k}$ (i=H,OH,P,OP for k=a; i=H,PH,PO,O for k=b; and i=HP,HO,P,O for k=c) are the corresponding dissipation coefficients including specific irreversible information. It can be seen from Equations (2)–(4) when $t_{i}^{k}\to \infty$ the reversible condition can be recovered.

It is worth stressing the importance of reversible entropy changes inside the combined models of three-terminal heat pump and heat transformer. For two-terminal low-dissipation thermodynamic devices, the value of reversible entropy is, to some extent, insignificant and usually regarded as a factor which makes the performance parameters dimensionless [12,17]. Nevertheless, for the three-terminal combined models, the connection and matching between two subsystems have great influence on the performance characteristics of the overall system. Therefore, by considering the practical meanings of reversible entropy changes [17,44], the parameters indicating the size ratio of heat pump to heat engine for models *A*, *B* and *C* are, respectively, introduced as

(5) $$C_{a,thp}=\frac{\Delta S_{hp}^{a}}{\Delta S_{he}^{a}}=\frac{T_{H}(1-\frac{\sigma_{H}^{a}}{t_{H}^{a}})-T_{O}(1+\frac{\sigma_{OH}^{a}}{t_{OH}^{a}})}{T_{P}(1+\frac{\sigma_{P}^{a}}{t_{P}^{a}})-T_{O}(1-\frac{\sigma_{OP}^{a}}{t_{OP}^{a}})},$$

(6) $$C_{b,thp}=\frac{\Delta S_{hp}^{b}}{\Delta S_{he}^{b}}=\frac{T_{H}(1-\frac{\sigma_{H}^{b}}{t_{H}^{b}})-T_{P}(1+\frac{\sigma_{PH}^{b}}{t_{PH}^{b}})}{T_{P}(1+\frac{\sigma_{PO}^{b}}{t_{PO}^{b}})-T_{O}(1-\frac{\sigma_{O}^{b}}{t_{O}^{b}})},$$

and

(7) $$C_{c,thp}=\frac{\Delta S_{hp}^{c}}{\Delta S_{he}^{c}}=\frac{T_{H}(1-\frac{\sigma_{HP}^{c}}{t_{HP}^{c}})-T_{P}(1+\frac{\sigma_{P}^{c}}{t_{P}^{c}})}{T_{H}(1+\frac{\sigma_{HO}^{c}}{t_{HO}^{c}})-T_{O}(1-\frac{\sigma_{O}^{c}}{t_{O}^{c}})}.$$

In Equations (5)–(7), the second equations are derived according to the law of energy conservation, i.e., $Q_{H}^{a}-Q_{OH}^{a}=Q_{P}^{a}-Q_{OP}^{a}$, $Q_{H}^{b}-Q_{PH}^{b}=Q_{PO}^{b}-Q_{O}^{b}$ and $Q_{HP}^{c}-Q_{P}^{c}=Q_{HO}^{c}-Q_{O}^{c}$. The size ratios for models *A*, *B* and *C* at reversible limit can be directly derived from Equations (5)–(7) as $C_{a,thp}^{r}=(T_{H}-T_{O})/(T_{P}-T_{O})$, $C_{b,thp}^{r}=(T_{H}-T_{P})/(T_{P}-T_{O})$ and $C_{c,thp}^{r}=(T_{H}-T_{P})/(T_{H}-T_{O})$ by setting $t_{i}^{k}\to \infty$. In addition, the time duration of the adiabatic process is usually assumed to be negligible comparing to heat exchanging process. Consequently, the heating loads and COPs of the models *A, B* and *C* can be expressed as

(8) $$R_{a,thp}=\frac{Q_{P}^{a}}{\tau_{a,thp}}=\frac{T_{P}\Delta S_{hp}^{a}(1+\frac{\sigma_{P}^{a}}{t_{P}^{a}})}{t_{H}^{a}+t_{OH}^{a}+t_{P}^{a}+t_{OP}^{a}}$$

(9) $$R_{b,thp}=\frac{Q_{PH}^{b}+Q_{PO}^{b}}{\tau_{b,thp}}=\frac{T_{P}\Delta S_{he}^{b}(1+\frac{\sigma_{PH}^{b}}{t_{PH}^{b}})+T_{P}\Delta S_{hp}^{b}(1+\frac{\sigma_{PO}^{b}}{t_{PO}^{b}})}{t_{H}^{b}+t_{PH}^{b}+t_{PO}^{b}+t_{O}^{b}},$$

(10) $$R_{c,thp}=\frac{Q_{P}^{c}}{\tau_{c,thp}}=\frac{T_{P}\Delta S_{he}^{c}(1+\frac{\sigma_{P}^{c}}{t_{P}^{c}})}{t_{P}^{c}+t_{HP}^{c}+t_{HO}^{c}+t_{O}^{c}},$$

(11) $$\psi_{a,thp}=\frac{Q_{P}^{a}}{Q_{H}^{a}}=\eta_{a,thp}\varepsilon_{a,thp}=C_{a,thp}\frac{T_{P}(1+\frac{\sigma_{P}^{a}}{t_{P}^{a}})}{T_{H}(1-\frac{\sigma_{H}^{a}}{t_{H}^{a}})}$$

(12) $$\psi_{b,thp}=\frac{Q_{PH}^{b}+Q_{PO}^{b}}{Q_{H}^{b}}=1+\eta_{b,thp}(\varepsilon_{b,thp}-1)=1+C_{b,thp}\frac{T_{O}(1-\frac{\sigma_{O}^{b}}{t_{O}^{b}})}{T_{H}(1-\frac{\sigma_{H}^{b}}{t_{H}^{b}})},$$

and

(13) $$\psi_{c,thp}=\frac{Q_{P}^{c}}{Q_{HP}^{c}-Q_{HO}^{c}}=\frac{1-\eta_{c,thp}}{1-\eta_{c,thp}\varepsilon_{c,thp}}=\frac{T_{P}(1+\frac{\sigma_{P}^{c}}{t_{P}^{c}})}{T_{H}(1-\frac{\sigma_{HP}^{c}}{t_{HP}^{c}})-C_{c,thp}T_{H}(1+\frac{\sigma_{HO}^{c}}{t_{HO}^{c}})},$$

respectively, where $\eta_{a,thp}=W_{a,thp}/Q_{H}^{a}$, $\varepsilon_{a,thp}=Q_{P}^{a}/W_{a,thp}$, $\eta_{b,thp}=W_{b,thp}/Q_{H}^{b}$, $\varepsilon_{b,thp}=Q_{PO}^{b}/W_{b,thp}$, $\eta_{c,thp}=W_{c,thp}/Q_{HP}^{c}$ and $\varepsilon_{c,thp}=Q_{HO}^{c}/W_{c,thp}$ are the efficiency of Carnot heat engine and the COP of the Carnot heat pump for models *A*, *B* and *C*, respectively. $W_{a,thp}=Q_{H}^{a}-Q_{OH}^{a}=Q_{P}^{a}-Q_{OP}^{a}$, $W_{b,thp}=Q_{H}^{b}-Q_{PH}^{b}=Q_{PO}^{b}-Q_{O}^{b}$ and $W_{c,thp}=Q_{HP}^{c}-Q_{P}^{c}=Q_{HO}^{c}-Q_{O}^{c}$ are the work transmitted between two subsystems in the models *A*, *B* and *C*, respectively.

### 2.2. Low-Dissipation Three-Terminal Heat Transformer

Likewise, based on the low-dissipation assumption, when the three-terminal heat transformer is constructed as models *A*, *B* and *C* (shown by Figure 3a–c, respectively), the heats exchanged between the system and the three heat reservoirs in four heat transferring processes can be expressed as [7]

(14) $$\left\{ \begin{array}{l} Q_{H}^{a}=Q_{Hr}^{a}(1-\frac{\sigma_{H}^{a}}{t_{H}^{a}})=T_{H}\Delta S_{he}^{a}(1-\frac{\sigma_{H}^{a}}{t_{H}^{a}})\\ Q_{OH}^{a}=Q_{OHr}^{a}(1+\frac{\sigma_{OH}^{a}}{t_{OH}^{a}})=T_{O}\Delta S_{he}^{a}(1+\frac{\sigma_{OH}^{a}}{t_{OH}^{a}})\\ Q_{OP}^{a}=Q_{OPr}^{a}(1-\frac{\sigma_{OP}^{a}}{t_{OP}^{a}})=T_{O}\Delta S_{hp}^{a}(1-\frac{\sigma_{OP}^{a}}{t_{OP}^{a}})\\ Q_{P}^{a}=Q_{Pr}^{a}(1+\frac{\sigma_{P}^{a}}{t_{P}^{a}})=T_{P}\Delta S_{hp}^{a}(1+\frac{\sigma_{P}^{a}}{t_{P}^{a}}) \end{array}\right.,$$

(15) $$\left\{ \begin{array}{l} Q_{P}^{b}=Q_{Pr}^{b}(1+\frac{\sigma_{P}^{b}}{t_{P}^{b}})=T_{P}\Delta S_{hp}^{b}(1+\frac{\sigma_{P}^{b}}{t_{P}^{b}})\\ Q_{HP}^{b}=Q_{HPr}^{b}(1-\frac{\sigma_{HP}^{b}}{t_{HP}^{b}})=T_{H}\Delta S_{hp}^{b}(1-\frac{\sigma_{HP}^{b}}{t_{HP}^{b}})\\ Q_{HO}^{b}=Q_{HOr}^{b}(1-\frac{\sigma_{HO}^{b}}{t_{HO}^{b}})=T_{H}\Delta S_{he}^{b}(1-\frac{\sigma_{HO}^{b}}{t_{HO}^{b}})\\ Q_{O}^{b}=Q_{Or}^{b}(1+\frac{\sigma_{O}^{b}}{t_{O}^{b}})=T_{O}\Delta S_{he}^{b}(1+\frac{\sigma_{O}^{b}}{t_{O}^{b}}) \end{array}\right.,$$

and

(16) $$\left\{ \begin{array}{l} Q_{PO}^{c}=Q_{POr}^{c}(1-\frac{\sigma_{PO}^{c}}{t_{PO}^{c}})=T_{P}\Delta S_{he}^{c}(1-\frac{\sigma_{PO}^{c}}{t_{PO}^{c}})\\ Q_{PH}^{c}=Q_{PHr}^{c}(1+\frac{\sigma_{PH}^{c}}{t_{PH}^{c}})=T_{P}\Delta S_{hp}^{c}(1+\frac{\sigma_{PH}^{c}}{t_{PH}^{c}})\\ Q_{H}^{c}=Q_{Hr}^{c}(1-\frac{\sigma_{H}^{c}}{t_{H}^{c}})=T_{H}\Delta S_{hp}^{c}(1-\frac{\sigma_{H}^{c}}{t_{H}^{c}})\\ Q_{O}^{c}=Q_{Or}^{c}(1+\frac{\sigma_{O}^{c}}{t_{O}^{c}})=T_{O}\Delta S_{he}^{c}(1+\frac{\sigma_{O}^{c}}{t_{O}^{c}}) \end{array}\right.,$$

respectively, where $Q_{ir}^{k}$ (i=H,OH,OP,P for k=a;  i=P,HP,HO,O  for k=b; and i=PO,PH,H,O for k=c) are the exchanged heats in the four heat transferring processes under reversible limit, $\Delta S_{j}^{k}$ (j=he,hp and k=a,b,c) are the corresponding reversible entropy changes, $t_{i}^{k}$ (i=H,OH,OP,P for k=a;  i=P,HP,HO,O  for k=b; and i=PO,PH,H,O for k=c) are the time durations of four heat exchanging processes and $\sigma_{i}^{k}$ (i=H,OH,OP,P for k=a;  i=P,HP,HO,O  for k=b; and i=PO,PH,H,O for k=c) are the corresponding dissipation coefficients including specific irreversible information.

Similarly, the size ratios for models *A*, *B* and *C* of the three-terminal heat transformer are given by

(17) $$D_{a,tht}=\frac{\Delta S_{hp}^{a}}{\Delta S_{he}^{a}}=\frac{T_{H}(1-\frac{\sigma_{H}^{a}}{t_{H}^{a}})-T_{O}(1+\frac{\sigma_{OH}^{a}}{t_{OH}^{a}})}{T_{P}(1+\frac{\sigma_{P}^{a}}{t_{P}^{a}})-T_{O}(1-\frac{\sigma_{OP}^{a}}{t_{OP}^{a}})},$$

(18) $$D_{b,tht}=\frac{\Delta S_{hp}^{b}}{\Delta S_{he}^{b}}=\frac{T_{H}(1-\frac{\sigma_{HO}^{b}}{t_{HO}^{b}})-T_{O}(1+\frac{\sigma_{O}^{b}}{t_{O}^{b}})}{T_{P}(1+\frac{\sigma_{P}^{b}}{t_{P}^{b}})-T_{H}(1-\frac{\sigma_{HP}^{b}}{t_{HP}^{b}})},$$

and

(19) $$D_{c,tht}=\frac{\Delta S_{hp}^{c}}{\Delta S_{he}^{c}}=\frac{T_{P}(1-\frac{\sigma_{PO}^{c}}{t_{PO}^{c}})-T_{O}(1+\frac{\sigma_{O}^{c}}{t_{O}^{c}})}{T_{P}(1+\frac{\sigma_{PH}^{c}}{t_{PH}^{c}})-T_{H}(1-\frac{\sigma_{H}^{c}}{t_{H}^{c}})}.$$

Noted from Equations (14)–(19) that the reversible regime can be approached in the limit of infinite time. In addition, the reversible size ratios for models *A*, *B* and *C* can be obtained as $D_{a,tht}^{r}=(T_{H}-T_{O})/(T_{P}-T_{O})$, $D_{b,tht}^{r}=(T_{H}-T_{O})/(T_{P}-T_{H})$ and $D_{c,tht}^{r}=(T_{P}-T_{O})/(T_{P}-T_{H})$.

When the time duration of the adiabatic process is assumed to be neglected, the expressions of heating load and COP for models *A*, *B* and *C* can be derived as

(20) $$R_{a,tht}=\frac{Q_{P}^{a}}{\tau_{a,tht}}=\frac{T_{P}\Delta S_{hp}^{a}(1+\frac{\sigma_{P}^{a}}{t_{P}^{a}})}{t_{H}^{a}+t_{OH}^{a}+t_{OP}^{a}+t_{P}^{a}},$$

(21) $$R_{b,tht}=\frac{Q_{P}^{b}}{\tau_{b,tht}}=\frac{T_{P}\Delta S_{hp}^{b}(1+\frac{\sigma_{P}^{b}}{t_{P}^{b}})}{t_{P}^{b}+t_{HO}^{b}+t_{HP}^{b}+t_{O}^{b}},$$

(22) $$R_{c,tht}=\frac{Q_{PH}^{c}-Q_{PO}^{c}}{\tau_{c,tht}}=\frac{T_{P}\Delta S_{hp}^{c}(1+\frac{\sigma_{PH}^{c}}{t_{PH}^{c}})-T_{P}\Delta S_{he}^{c}(1-\frac{\sigma_{PO}^{c}}{t_{PO}^{c}})}{t_{H}^{c}+t_{PH}^{c}+t_{PO}^{c}+t_{O}^{c}},$$

(23) $$\psi_{a,tht}=\frac{Q_{P}^{a}}{Q_{H}^{a}}=\varepsilon_{a,tht}\eta_{a,tht}=D_{a,tht}\frac{T_{P}(1+\frac{\sigma_{P}^{a}}{t_{P}^{a}})}{T_{H}(1-\frac{\sigma_{H}^{a}}{t_{H}^{a}})},$$

(24) $$\psi_{b,tht}=\frac{Q_{P}^{b}}{Q_{HP}^{b}+Q_{HO}^{b}}={\left[1+\frac{1}{\varepsilon_{b,tht}}(\frac{1}{\eta_{b,tht}}-1)\right]}^{-1}=\frac{T_{P}D_{b,tht}(1+\frac{\sigma_{P}^{b}}{t_{P}^{b}})}{T_{H}D_{b,tht}(1-\frac{\sigma_{HP}^{b}}{t_{HP}^{b}})+T_{H}(1-\frac{\sigma_{HO}^{b}}{t_{HO}^{b}})},$$

and

(25) $$\psi_{c,tht}=\frac{Q_{PH}^{c}-Q_{PO}^{c}}{Q_{H}^{c}}=\frac{\varepsilon_{c,tht}\eta_{c,tht}-1}{\varepsilon_{c,tht}\eta_{c,tht}-\eta_{c,tht}}=1-\frac{1}{D_{c,tht}}\frac{T_{O}(1+\frac{\sigma_{O}^{c}}{t_{O}^{c}})}{T_{H}(1-\frac{\sigma_{H}^{c}}{t_{H}^{c}})},$$

respectively, where $\eta_{a,tht}=W_{a,tht}/Q_{H}^{a}$, $\varepsilon_{a,tht}=Q_{P}^{a}/W_{a,tht}$, $\eta_{b,tht}=W_{b,tht}/Q_{HO}^{b}$, $\varepsilon_{b,tht}=Q_{P}^{b}/W_{b,tht}$, $\eta_{c,tht}=W_{c,tht}/Q_{PO}^{c}$ and $\varepsilon_{c,tht}=Q_{PH}^{c}/W_{c,tht}$ are the efficiency of Carnot heat engine and the COP of the Carnot heat pump for models *A*, *B* and *C* respectively. $W_{k,tht}$ (k=a,b,c) are the transferred work between two subsystems for models *A*, *B* and *C*.

## 3. Parametric Optimum Analyses

### 3.1. Optimal Coefficient of Performance and the Corresponding Parametric Optimizations for Three-Terminal Heat Pump

It can be seen from Equations (11)–(13), when both $\varepsilon_{k,thp}$ (k=a,b,c) and $\eta_{k,thp}$ (k=a,b,c) attain their maxima, $\psi_{k,thp}$ (k=a,b,c) are optimized. According to the models established above and the definitions of $\eta_{k,thp}$ (k=a,b,c), the relations

(26) $${\tilde{t}}_{H}^{a}=\left\{ \begin{array}{l} \frac{{\tilde{\sigma }}_{H}^{a}{\tilde{\tau }}_{he}^{a}-\sqrt{{\tilde{\sigma }}_{H}^{a}{\tilde{\tau }}_{he}^{a}[{\tilde{\tau }}_{he}^{a}-(2{\tilde{\sigma }}_{H}^{a}-1)](1-{\tilde{\sigma }}_{H}^{a})}}{2{\tilde{\sigma }}_{H}^{a}-1},\;{\tilde{\sigma }}_{H}^{a}\neq 0.5\\ \frac{1+2{\tilde{\tau }}_{he}^{a}}{4}\;\;\;\;\;\;\;\;\;\;\;\;\;\;\;\;\;\;\;\;\;\;\;\;\;\;\;\;\;\;\;\;\;\;\;\;\;\;\;\;\;,\;{\tilde{\sigma }}_{H}^{a}=0.5 \end{array}\right.,$$

(27) $${\tilde{t}}_{H}^{b}=\left\{ \begin{array}{l} \frac{{\tilde{\sigma }}_{H}^{b}{\tilde{\tau }}_{he}^{b}-\sqrt{{\tilde{\sigma }}_{H}^{b}{\tilde{\tau }}_{he}^{b}[{\tilde{\tau }}_{he}^{b}-(2{\tilde{\sigma }}_{H}^{b}-1)](1-{\tilde{\sigma }}_{H}^{b})}}{2{\tilde{\sigma }}_{H}^{b}-1},\;{\tilde{\sigma }}_{H}^{b}\neq 0.5\\ \frac{1+2{\tilde{\tau }}_{he}^{b}}{4}\;\;\;\;\;\;\;\;\;\;\;\;\;\;\;\;\;\;\;\;\;\;\;\;\;\;\;\;\;\;\;\;\;\;\;\;\;\;\;\;\;,\;{\tilde{\sigma }}_{H}^{b}=0.5 \end{array}\right.,$$

and

(28) $${\tilde{t}}_{HP}^{c}=\left\{ \begin{array}{l} \frac{{\tilde{\sigma }}_{HP}^{c}{\tilde{\tau }}_{he}^{c}-\sqrt{{\tilde{\sigma }}_{HP}^{c}{\tilde{\tau }}_{he}^{c}[{\tilde{\tau }}_{he}^{c}-(2{\tilde{\sigma }}_{HP}^{c}-1)](1-{\tilde{\sigma }}_{HP}^{c})}}{2{\tilde{\sigma }}_{HP}^{c}-1},{\tilde{\sigma }}_{HP}^{c}\neq 0.5\\ \frac{1+2{\tilde{\tau }}_{he}^{c}}{4}\;\;\;\;\;\;\;\;\;\;\;\;\;\;\;\;\;\;\;\;\;\;\;\;\;\;\;\;\;\;\;\;\;\;\;\;\;\;\;\;\;\;\;\;\;\;\;\;\;,\;{\tilde{\sigma }}_{HP}^{c}=0.5 \end{array}\right.$$

should be, respectively, satisfied to make $\eta_{k,thp}$ (k=a,b,c) maximum, where ${\tilde{T}}_{H}=T_{H}/T_{O}$, ${\tilde{\sigma }}_{H}^{a}=\sigma_{H}^{a}/(\sigma_{H}^{a}+\sigma_{OH}^{a})$, ${\tilde{\tau }}_{he}^{a}=(t_{H}^{a}+t_{OH}^{a})/(\sigma_{H}^{a}+\sigma_{OH}^{a})$ and ${\tilde{t}}_{H}^{a}=t_{H}^{a}/(\sigma_{H}^{a}+\sigma_{OH}^{a})$; ${\tilde{\sigma }}_{H}^{b}=\sigma_{H}^{b}/(\sigma_{H}^{b}+\sigma_{PH}^{b})$, ${\tilde{\tau }}_{he}^{b}=(t_{H}^{b}+t_{PH}^{b})/(\sigma_{H}^{b}+\sigma_{PH}^{b})$ and ${\tilde{t}}_{H}^{b}=t_{H}^{b}/(\sigma_{H}^{b}+\sigma_{PH}^{b})$; ${\tilde{\sigma }}_{HP}^{c}=\sigma_{HP}^{c}/(\sigma_{P}^{c}+\sigma_{HP}^{c})$, ${\tilde{\tau }}_{he}^{c}=(t_{P}^{c}+t_{HP}^{c})/(\sigma_{P}^{c}+\sigma_{HP}^{c})$ and ${\tilde{t}}_{HP}^{c}=t_{HP}^{c}/(\sigma_{P}^{c}+\sigma_{HP}^{c})$.

Similarly, according to the definitions of $\varepsilon_{k,thp}$ (k=a,b,c), one can prove that the $\varepsilon_{k,thp}$ (k=a,b,c) are optimum at the conditions of

(29) $${\tilde{t}}_{OP}^{a}=\left\{ \begin{array}{l} \frac{{\tilde{\sigma }}_{OP}^{a}{\tilde{\tau }}_{hp}^{a}-\sqrt{{\tilde{\sigma }}_{OP}^{a}{\tilde{\tau }}_{hp}^{a}[{\tilde{\tau }}_{hp}^{a}-(2{\tilde{\sigma }}_{OP}^{a}-1)](1-{\tilde{\sigma }}_{OP}^{a})}}{2{\tilde{\sigma }}_{OP}^{a}-1},\;{\tilde{\sigma }}_{OP}^{a}\neq 0.5\\ \frac{1+2{\tilde{\tau }}_{hp}^{a}}{4}\;\;\;\;\;\;\;\;\;\;\;\;\;\;\;\;\;\;\;\;\;\;\;\;\;\;\;\;\;\;\;\;\;\;\;\;\;\;\;\;\;\;\;\;\;\;\;\;,\;{\tilde{\sigma }}_{OP}^{a}=0.5 \end{array}\right.,$$

(30) $${\tilde{t}}_{O}^{b}=\left\{ \begin{array}{l} \frac{{\tilde{\sigma }}_{O}^{b}{\tilde{\tau }}_{hp}^{b}-\sqrt{{\tilde{\sigma }}_{O}^{b}{\tilde{\tau }}_{hp}^{b}[{\tilde{\tau }}_{hp}^{b}-(2{\tilde{\sigma }}_{O}^{b}-1)](1-{\tilde{\sigma }}_{O}^{b})}}{2{\tilde{\sigma }}_{O}^{b}-1},\;{\tilde{\sigma }}_{O}^{b}\neq 0.5\\ \frac{1+2{\tilde{\tau }}_{hp}^{b}}{4}\;\;\;\;\;\;\;\;\;\;\;\;\;\;\;\;\;\;\;\;\;\;\;\;\;\;\;\;\;\;\;\;\;\;\;\;\;\;\;\;\;,\;{\tilde{\sigma }}_{O}^{b}=0.5 \end{array}\right.,$$

and

(31) $${\tilde{t}}_{O}^{c}=\left\{ \begin{array}{l} \frac{{\tilde{\sigma }}_{O}^{c}{\tilde{\tau }}_{hp}^{c}-\sqrt{{\tilde{\sigma }}_{O}^{c}{\tilde{\tau }}_{hp}^{c}[{\tilde{\tau }}_{hp}^{c}-(2{\tilde{\sigma }}_{O}^{c}-1)](1-{\tilde{\sigma }}_{O}^{c})}}{2{\tilde{\sigma }}_{O}^{c}-1},\;{\tilde{\sigma }}_{O}^{c}\neq 0.5\\ \frac{1+2{\tilde{\tau }}_{hp}^{c}}{4}\;\;\;\;\;\;\;\;\;\;\;\;\;\;\;\;\;\;\;\;\;\;\;\;\;\;\;\;\;\;\;\;\;\;\;\;\;\;\;\;\;,\;{\tilde{\sigma }}_{O}^{c}=0.5 \end{array}\right.,$$

respectively, where ${\tilde{\sigma }}_{OP}^{a}=\sigma_{OP}^{a}/(\sigma_{P}^{a}+\sigma_{OP}^{a})$, ${\tilde{\tau }}_{hp}^{a}=(t_{P}^{a}+t_{OP}^{a})/(\sigma_{P}^{a}+\sigma_{OP}^{a})$ and ${\tilde{t}}_{OP}^{a}=t_{OP}^{a}/(\sigma_{P}^{a}+\sigma_{OP}^{a})$; ${\tilde{\sigma }}_{O}^{b}=\sigma_{O}^{b}/(\sigma_{O}^{b}+\sigma_{PO}^{b})$, ${\tilde{\tau }}_{hp}^{b}=(t_{O}^{b}+t_{PO}^{b})/(\sigma_{O}^{b}+\sigma_{PO}^{b})$ and ${\tilde{t}}_{O}^{b}=t_{O}^{b}/(\sigma_{O}^{b}+\sigma_{PO}^{b})$; ${\tilde{\sigma }}_{O}^{c}=\sigma_{O}^{c}/(\sigma_{HO}^{c}+\sigma_{O}^{c})$, ${\tilde{\tau }}_{hp}^{c}=(t_{HO}^{c}+t_{O}^{c})/(\sigma_{HO}^{c}+\sigma_{O}^{c})$ and ${\tilde{t}}_{O}^{c}=t_{O}^{c}/(\sigma_{HO}^{c}+\sigma_{O}^{c})$.

### 3.2. Optimal Coefficient of Performance and the Corresponding Parametric Optimizations for Three-Terminal Heat Transformer

According to Equations (23)–(25), one can realize that $\psi_{k,tht}$ (k=a,b,c) are optimized at the conditions making both $\varepsilon_{k,tht}$ (k=a,b,c) and $\eta_{k,tht}$ (k=a,b,c) maximum.

Based on the definitions of $\eta_{k,tht}$ (k=a,b,c), one can find when the relations

(32) $${\tilde{t}}_{H}^{a}=\left\{ \begin{array}{l} \frac{{\tilde{\sigma }}_{H}^{a}{\tilde{\tau }}_{he}^{a}-\sqrt{{\tilde{\sigma }}_{H}^{a}{\tilde{\tau }}_{he}^{a}[{\tilde{\tau }}_{he}^{a}-(2{\tilde{\sigma }}_{H}^{a}-1)](1-{\tilde{\sigma }}_{H}^{a})}}{2{\tilde{\sigma }}_{H}^{a}-1},\;{\tilde{\sigma }}_{H}^{a}\neq 0.5\\ \frac{1+2{\tilde{\tau }}_{he}^{a}}{4}\;\;\;\;\;\;\;\;\;\;\;\;\;\;\;\;\;\;\;\;\;\;\;\;\;\;\;\;\;\;\;\;\;\;\;\;\;\;\;\;\;,\;{\tilde{\sigma }}_{H}^{a}=0.5 \end{array}\right.,$$

(33) $${\tilde{t}}_{HO}^{b}=\left\{ \begin{array}{l} \frac{{\tilde{\sigma }}_{HO}^{b}{\tilde{\tau }}_{he}^{b}-\sqrt{{\tilde{\sigma }}_{HO}^{b}{\tilde{\tau }}_{he}^{b}[{\tilde{\tau }}_{he}^{b}-(2{\tilde{\sigma }}_{HO}^{b}-1)](1-{\tilde{\sigma }}_{HO}^{b})}}{2{\tilde{\sigma }}_{HO}^{b}-1},\;{\tilde{\sigma }}_{HO}^{b}\neq 0.5\\ \frac{1+2{\tilde{\tau }}_{he}^{b}}{4}\;\;\;\;\;\;\;\;\;\;\;\;\;\;\;\;\;\;\;\;\;\;\;\;\;\;\;\;\;\;\;\;\;\;\;\;\;\;\;\;\;\;\;\;\;\;\;\;\;,\;{\tilde{\sigma }}_{HO}^{b}=0.5 \end{array}\right.,$$

and

(34) $${\tilde{t}}_{PO}^{c}=\left\{ \begin{array}{l} \frac{{\tilde{\sigma }}_{PO}^{c}{\tilde{\tau }}_{he}^{c}-\sqrt{{\tilde{\sigma }}_{PO}^{c}{\tilde{\tau }}_{he}^{c}[{\tilde{\tau }}_{he}^{c}-(2{\tilde{\sigma }}_{PO}^{c}-1)](1-{\tilde{\sigma }}_{PO}^{c})}}{2{\tilde{\sigma }}_{PO}^{c}-1},\;{\tilde{\sigma }}_{PO}^{c}\neq 0.5\\ \frac{1+2{\tilde{\tau }}_{he}^{c}}{4}\;\;\;\;\;\;\;\;\;\;\;\;\;\;\;\;\;\;\;\;\;\;\;\;\;\;\;\;\;\;\;\;\;\;\;\;\;\;\;\;\;\;\;\;\;\;\;,\;{\tilde{\sigma }}_{PO}^{c}=0.5 \end{array}\right.$$

are, respectively, satisfied, $\eta_{k,tht}$ (k=a,b,c) reach their maxima. In Equations (32)–(34), ${\tilde{\sigma }}_{H}^{a}=\sigma_{H}^{a}/(\sigma_{H}^{a}+\sigma_{OH}^{a})$, ${\tilde{\tau }}_{he}^{a}=(t_{H}^{a}+t_{OH}^{a})/(\sigma_{H}^{a}+\sigma_{OH}^{a})$ and ${\tilde{t}}_{H}^{a}=t_{H}^{a}/(\sigma_{H}^{a}+\sigma_{OH}^{a})$; ${\tilde{\sigma }}_{HO}^{b}=\sigma_{HO}^{b}/(\sigma_{HO}^{b}+\sigma_{O}^{b})$, ${\tilde{\tau }}_{he}^{b}=(t_{HO}^{b}+t_{O}^{b})/(\sigma_{HO}^{b}+\sigma_{O}^{b})$ and ${\tilde{t}}_{HO}^{b}=t_{HO}^{b}/(\sigma_{O}^{b}+\sigma_{HO}^{b})$; ${\tilde{\sigma }}_{PO}^{c}=\sigma_{PO}^{c}/(\sigma_{O}^{c}+\sigma_{PO}^{c})$, ${\tilde{\tau }}_{he}^{c}=(t_{O}^{c}+t_{PO}^{c})/(\sigma_{O}^{c}+\sigma_{PO}^{c})$ and ${\tilde{t}}_{PO}^{c}=t_{PO}^{c}/(\sigma_{O}^{c}+\sigma_{PO}^{c})$.

Likewise, the optimum conditions for maximizing $\varepsilon_{k,tht}$ (k=a,b,c) can be derived as

(35) $${\tilde{t}}_{OP}^{a}=\left\{ \begin{array}{l} \frac{{\tilde{\sigma }}_{OP}^{a}{\tilde{\tau }}_{hp}^{a}-\sqrt{{\tilde{\sigma }}_{OP}^{a}{\tilde{\tau }}_{hp}^{a}[{\tilde{\tau }}_{hp}^{a}-(2{\tilde{\sigma }}_{OP}^{a}-1)](1-{\tilde{\sigma }}_{OP}^{a})}}{2{\tilde{\sigma }}_{OP}^{a}-1},\;{\tilde{\sigma }}_{OP}^{a}\neq 0.5\\ \frac{1+2{\tilde{\tau }}_{hp}^{a}}{4}\;\;\;\;\;\;\;\;\;\;\;\;\;\;\;\;\;\;\;\;\;\;\;\;\;\;\;\;\;\;\;\;\;\;\;\;\;\;\;\;\;\;\;\;\;\;\;\;\;\;,\;{\tilde{\sigma }}_{OP}^{a}=0.5 \end{array}\right.,$$

(36) $${\tilde{t}}_{HP}^{b}=\left\{ \begin{array}{l} \frac{{\tilde{\sigma }}_{HP}^{b}{\tilde{\tau }}_{hp}^{b}-\sqrt{{\tilde{\sigma }}_{HP}^{b}{\tilde{\tau }}_{hp}^{b}[{\tilde{\tau }}_{hp}^{b}-(2{\tilde{\sigma }}_{HP}^{b}-1)](1-{\tilde{\sigma }}_{HP}^{b})}}{2{\tilde{\sigma }}_{HP}^{b}-1},\;{\tilde{\sigma }}_{HP}^{b}\neq 0.5\\ \frac{1+2{\tilde{\tau }}_{hp}^{b}}{4}\;\;\;\;\;\;\;\;\;\;\;\;\;\;\;\;\;\;\;\;\;\;\;\;\;\;\;\;\;\;\;\;\;\;\;\;\;\;\;\;\;\;\;\;\;\;\;\;\;\;,\;{\tilde{\sigma }}_{HP}^{b}=0.5 \end{array}\right.,$$

and

(37) $${\tilde{t}}_{H}^{c}=\left\{ \begin{array}{l} \frac{{\tilde{\sigma }}_{H}^{c}{\tilde{\tau }}_{hp}^{c}-\sqrt{{\tilde{\sigma }}_{H}^{c}{\tilde{\tau }}_{hp}^{c}[{\tilde{\tau }}_{hp}^{c}-(2{\tilde{\sigma }}_{H}^{c}-1)](1-{\tilde{\sigma }}_{H}^{c})}}{2{\tilde{\sigma }}_{H}^{c}-1},\;{\tilde{\sigma }}_{H}^{c}\neq 0.5\\ \frac{1+2{\tilde{\tau }}_{hp}^{c}}{4}\;\;\;\;\;\;\;\;\;\;\;\;\;\;\;\;\;\;\;\;\;\;\;\;\;\;\;\;\;\;\;\;\;\;\;\;\;\;\;\;\;,\;{\tilde{\sigma }}_{H}^{c}=0.5 \end{array}\right.,$$

where ${\tilde{\sigma }}_{OP}^{a}=\sigma_{OP}^{a}/(\sigma_{P}^{a}+\sigma_{OP}^{a})$, ${\tilde{\tau }}_{hp}^{a}=(t_{P}^{a}+t_{OP}^{a})/(\sigma_{P}^{a}+\sigma_{OP}^{a})$ and ${\tilde{t}}_{OP}^{a}=t_{OP}^{a}/(\sigma_{P}^{a}+\sigma_{OP}^{a})$; ${\tilde{\sigma }}_{HP}^{b}=\sigma_{HP}^{b}/(\sigma_{P}^{b}+\sigma_{HP}^{b})$, ${\tilde{\tau }}_{hp}^{b}=(t_{P}^{b}+t_{HP}^{b})/(\sigma_{P}^{b}+\sigma_{HP}^{b})$ and ${\tilde{t}}_{HP}^{b}=t_{HP}^{b}/(\sigma_{P}^{b}+\sigma_{HP}^{b})$; ${\tilde{\sigma }}_{H}^{c}=\sigma_{H}^{c}/(\sigma_{H}^{c}+\sigma_{PH}^{c})$, ${\tilde{\tau }}_{hp}^{c}=(t_{H}^{c}+t_{PH}^{c})/(\sigma_{H}^{c}+\sigma_{PH}^{c})$ and ${\tilde{t}}_{H}^{c}=t_{H}^{c}/(\sigma_{H}^{c}+\sigma_{PH}^{c})$.

## 4. Results and Discussion

Note from the expressions of heating load and COP for both three-terminal heat pump and heat transformer that the size ratio between two subsystems has significant influences on the performance. However, for given operating temperatures, the values of size ratio under reversible limit for different constructions of system are not the same. In other words, different constructions of system with the same value of size ratio correspond to different irreversibilities. As a consequence, in order to evaluate and compare the performances of different constructions of system at the same level of irreversibility for both three-terminal heat pump and heat transformer, a parameter defined as $\alpha =C_{a,thp}^{}/C_{a,thp}^{r}=C_{b,thp}^{}/C_{b,thp}^{r}=C_{c,thp}^{}/C_{c,thp}^{r}=D_{a,tht}^{}/D_{a,tht}^{r}=D_{b,tht}^{}/D_{b,tht}^{r}=D_{c,tht}^{}/D_{c,tht}^{r}$ measuring the deviation from the reversible limit of the system is introduced. The value of α is located in the region of 0<α≤1. In addition, for the convenience of discussion, dimensionless heating loads will be introduced in the following discussion. For different constructions of the three-terminal heat pump and heat transformer, the dimensionless heating loads are given by

(38) $${\tilde{R}}_{a,thp}=R_{a,thp}\frac{\sigma_{H}^{a}+\sigma_{OH}^{a}}{T_{O}\Delta S_{he}^{a}}=\frac{C_{a,thp}{\tilde{T}}_{P}(1+\frac{1-{\tilde{\sigma }}_{OP}^{a}}{{\tilde{\tau }}_{hp}^{a}-{\tilde{t}}_{OP}^{a}})}{{\tilde{\tau }}_{he}^{a}+{\tilde{\tau }}_{hp}^{a}\frac{1-\beta_{a,thp}}{\beta_{a,thp}}},$$

(39) $${\tilde{R}}_{b,thp}=R_{b,thp}\frac{\sigma_{H}^{b}+\sigma_{PH}^{b}}{T_{O}\Delta S_{he}^{b}}=\frac{{\tilde{T}}_{P}[(1+\frac{1-{\tilde{\sigma }}_{H}^{b}}{{\tilde{\tau }}_{he}^{b}-{\tilde{t}}_{H}^{b}})+C_{b,thp}(1+\frac{1-{\tilde{\sigma }}_{O}^{b}}{{\tilde{\tau }}_{hp}^{b}-{\tilde{t}}_{O}^{b}})]}{{\tilde{\tau }}_{he}^{b}+{\tilde{\tau }}_{hp}^{b}\frac{1-\beta_{b,thp}}{\beta_{b,thp}}},$$

(40) $${\tilde{R}}_{c,thp}=R_{c,thp}\frac{\sigma_{HP}^{c}+\sigma_{P}^{c}}{T_{O}\Delta S_{he}^{c}}={\tilde{T}}_{P}\frac{1+\frac{1-{\tilde{\sigma }}_{HP}^{c}}{{\tilde{\tau }}_{he}^{c}-{\tilde{t}}_{HP}^{c}}}{{\tilde{\tau }}_{he}^{c}+{\tilde{\tau }}_{hp}^{c}\frac{1-\beta_{c,thp}}{\beta_{c,thp}}},$$

(41) $${\tilde{R}}_{a,tht}=R_{a,tht}\frac{\sigma_{OP}^{a}+\sigma_{P}^{a}}{T_{O}\Delta S_{hp}^{a}}=\frac{{\tilde{T}}_{P}(1+\frac{1-{\tilde{\sigma }}_{OP}^{a}}{{\tilde{\tau }}_{hp}-{\tilde{t}}_{OP}^{a}})}{{\tilde{\tau }}_{hp}^{a}+{\tilde{\tau }}_{he}^{a}\frac{1-\beta_{a,tht}}{\beta_{a,tht}}},$$

(42) $${\tilde{R}}_{b,tht}=R_{b,tht}\frac{\sigma_{P}^{b}+\sigma_{HP}^{b}}{T_{O}\Delta S_{hp}^{b}}=\frac{{\tilde{T}}_{P}(1+\frac{1-{\tilde{\sigma }}_{HP}^{b}}{{\tilde{\tau }}_{hp}^{b}-{\tilde{t}}_{HP}^{b}})}{{\tilde{\tau }}_{hp}^{b}+{\tilde{\tau }}_{he}^{b}\frac{1-\beta_{b,tht}}{\beta_{b,tht}}},$$

and

(43) $${\tilde{R}}_{c,tht}=R_{c,tht}\frac{\sigma_{H}^{c}+\sigma_{PH}^{c}}{T_{O}\Delta S_{hp}^{c}}=\frac{{\tilde{T}}_{P}(1+\frac{1-{\tilde{\sigma }}_{H}^{c}}{{\tilde{\tau }}_{hp}^{c}-{\tilde{t}}_{H}^{c}})-{\tilde{T}}_{P}\frac{1}{D_{c,tht}}(1-\frac{{\tilde{\sigma }}_{PO}^{c}}{{\tilde{t}}_{PO}^{c}})}{{\tilde{\tau }}_{hp}^{c}+{\tilde{\tau }}_{he}^{c}\frac{1-\beta_{c,tht}}{\beta_{c,tht}}},$$

where $\beta_{a,thp}=(\sigma_{H}^{a}+\sigma_{OH}^{a})/(\sigma_{H}^{a}+\sigma_{OH}^{a}+\sigma_{P}^{a}+\sigma_{OP}^{a})$, $\beta_{b,thp}=(\sigma_{H}^{b}+\sigma_{PH}^{b})/(\sigma_{H}^{b}+\sigma_{PH}^{b}+\sigma_{PO}^{b}+\sigma_{O}^{b})$, $\beta_{c,thp}=(\sigma_{HP}^{c}+\sigma_{P}^{c})/(\sigma_{HP}^{c}+\sigma_{P}^{c}+\sigma_{HO}^{c}+\sigma_{O}^{c})$, $\beta_{a,tht}=(\sigma_{OP}^{a}+\sigma_{P}^{a})/(\sigma_{H}^{a}+\sigma_{OH}^{a}+\sigma_{OP}^{a}+\sigma_{P}^{a})$, $\beta_{b,tht}=(\sigma_{P}^{b}+\sigma_{HP}^{b})/(\sigma_{P}^{b}+\sigma_{HO}^{b}+\sigma_{HP}^{b}+\sigma_{O}^{b})$, $\beta_{c,tht}=(\sigma_{H}^{c}+\sigma_{PH}^{c})/(\sigma_{H}^{c}+\sigma_{PH}^{c}+\sigma_{PO}^{c}+\sigma_{O}^{c})$.

It is worth mentioning that $\tilde{\sigma }$ (In Equations (26)–(37)) and β are two important parameters indicating the dissipative symmetry inside the individual subsystem and the dissipative symmetry between two subsystems, respectively, whose influences will be investigated in the following.

### 4.1. The Influence of α

**Figure 6:**
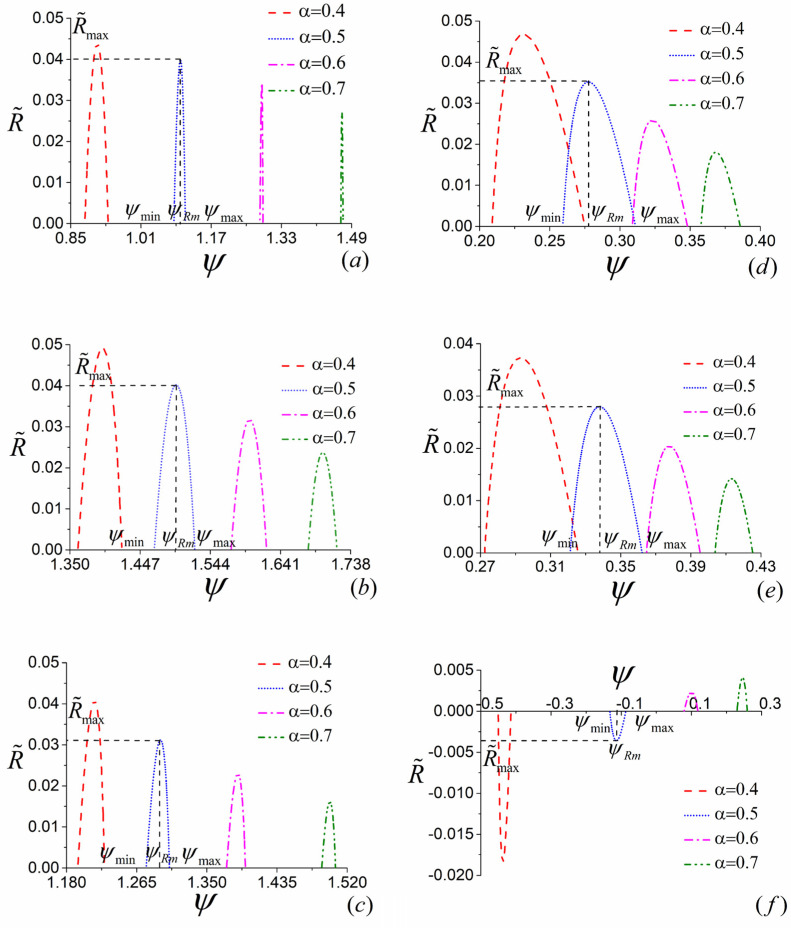
(**a**–**c**) The curves of optimized ψ varying with the corresponding $\tilde{R}$ with different values of α for the combined cycle models *A*, *B* and *C* of three-terminal heat pump, where ${\tilde{\sigma }}_{i}={\tilde{\sigma }}_{j}=0.3$ (i=H,j=OP for (**a**), i=O,j=H for (**b**), i=HP,j=O for (**c**)), ${\tilde{T}}_{H}=1.5$, ${\tilde{T}}_{P}=1.2$, $\beta_{k,thp}=0.5$ (k=a,b,c). (**d**–**f**) The curves of optimized ψ varying with the corresponding $\tilde{R}$ with different values of α for the combined cycle models *A*, *B* and *C* of three-terminal heat transformer, where ${\tilde{\sigma }}_{i}={\tilde{\sigma }}_{j}=0.3$ (i=H,j=OP for (**d**), i=HO,j=HP for (**e**) and i=H,j=PO for (**f**)), ${\tilde{T}}_{H}=1.2$, ${\tilde{T}}_{P}=1.5$, $\beta_{k,tht}=0.5$ (k=a,b,c).

By using Equations (5)–(7), (11)–(13), (26)–(31) and (38)–(40) and the numerical calculation conducted with Mathematica, the optimal relations between the COP and heating load of the low-dissipation three-terminal heat pump for various combined models and different values of α can be generated, as shown in Figure 6a–c. It can be seen from Figure 6a–c that, for all three various constructions of the three-terminal heat pump, the optimal relationship between ψ and $\tilde{R}$ is not monotonic and there exists an optimal value $\psi_{Rm}$ at which $\tilde{R}$ attains its maximum ${\tilde{R}}_{\max}$. When $\tilde{R}=0$, ψ has corresponding maximum value $\psi_{\max}$ and minimum value $\psi_{\min}$. For the given value of α, the three-terminal heat pump should be operated in the region of $\psi_{Rm}<\psi <\psi_{\max}$ at which the compromise between ψ and $\tilde{R}$ needs to be made according to the performance requirement. In addition, Figure 6a–c show that the values of COP for all three models increase with the increase of α, which is an expected result. More importantly, the COPs of models *B* and *C* are always larger than 1, while the COP of model *A* can be less than 1 when the value of α is small, namely, the deviation from the reversible limit is great. It is a reasonable result which can be explained as follows. According to Figure 2a–c, Equations (11)–(13) and the first law of thermodynamics, one has $\psi_{b,thp}=(Q_{PH}^{b}+Q_{PO}^{b})/Q_{H}^{b}=(Q_{PH}^{b}+W_{b,thp}+Q_{O}^{b})/Q_{H}^{b}>(Q_{PH}^{b}+W_{b,thp})/Q_{H}^{b}=1$ and $\psi_{c,thp}=Q_{P}^{c}/(Q_{HP}^{c}-Q_{HO}^{c})=Q_{P}^{c}/(Q_{HP}^{c}-W_{c,thp}-Q_{O}^{c})>Q_{P}^{c}/(Q_{HP}^{c}-W_{c,thp})=1$ for models *B* and *C*. Nevertheless, for model *A*, $\psi_{a,thp}=Q_{P}^{a}/Q_{H}^{a}=(W_{a,thp}+Q_{OP}^{a})/Q_{H}^{a}>W_{a,thp}/Q_{H}^{a}=\eta_{a,thp}$, which can be smaller than 1. It is necessary to point out that a three-terminal heat pump ($T_{H}>T_{P}$) with the COP less than 1 is meaningless in practice.

Similarly, Using Equations (17)–(19), (23)–(25), (32)–(37) and (41)–(43) and the numerical calculation conducted with Mathematica, one can obtain the optimal curves of the COP varying with heating load for different combined models of the low-dissipation three-terminal heat transformer with different values of α which are depicted by Figure 6d–f. It can be seen from Figure 6d–f that most of the characteristics of the curves are similar to Figure 6a–c, except the values of COP are less than 1. To be specific, for models *A* and *B*, the COPs are bounded by 0 and 1. Whereas the COP of model *C* can be less than 0 at some circumstances, as shown by Figure 6f. The reasonability of this characteristics can be realized from Figure 3a–c, Equations (23)–(25) and the first law of thermodynamics. For model *A*, $\psi_{a,tht}=Q_{P}^{a}/Q_{H}^{a}=(W_{a,tht}+Q_{OP}^{a})/Q_{H}^{a}<W_{a,tht}/Q_{H}^{a}=\eta_{a,tht}$. Considering the values of $Q_{P}^{a}$ and $Q_{H}^{a}$ are both not less than 0, one can deduce that $\psi_{a,tht}$ should be located in the region of $0<\psi_{a,tht}<1$. For model *B*, $\psi_{b,tht}=Q_{P}^{b}/(Q_{HO}^{b}+Q_{HP}^{b})=(W_{b,tht}+Q_{HP}^{b})/(Q_{HO}^{b}+Q_{HP}^{b})$ whose value is bounded in the region of $0<\psi_{b,tht}<1$ by considering that $Q_{P}^{b}$, $Q_{HO}^{b}$ and $Q_{HP}^{b}$ are all not less than 0 and $Q_{HO}^{b}$ is greater than $W_{b,tht}$. For model *C*, $\psi_{c,tht}=(Q_{PH}^{c}-Q_{PO}^{c})/Q_{H}^{c}=(Q_{PH}^{c}-Q_{PO}^{c})/(Q_{PH}^{c}-W_{c,tht})$. Considering $Q_{PO}^{c}>W_{c,tht}$, one can deduce that $\psi_{c,tht}<1$. In addition, the value of $Q_{PH}^{c}$ is only limited by $Q_{PH}^{c}\geq W_{c,tht}$ which could be less than the value of $Q_{PO}^{c}$; therefore, $\psi_{c,tht}$ can be negative at some circumstances. It is worth noting that a three-terminal heat transformer ($T_{H}<T_{P}$) with negative COP is meaningless.

### 4.2. The Influence of β

**Figure 7:**
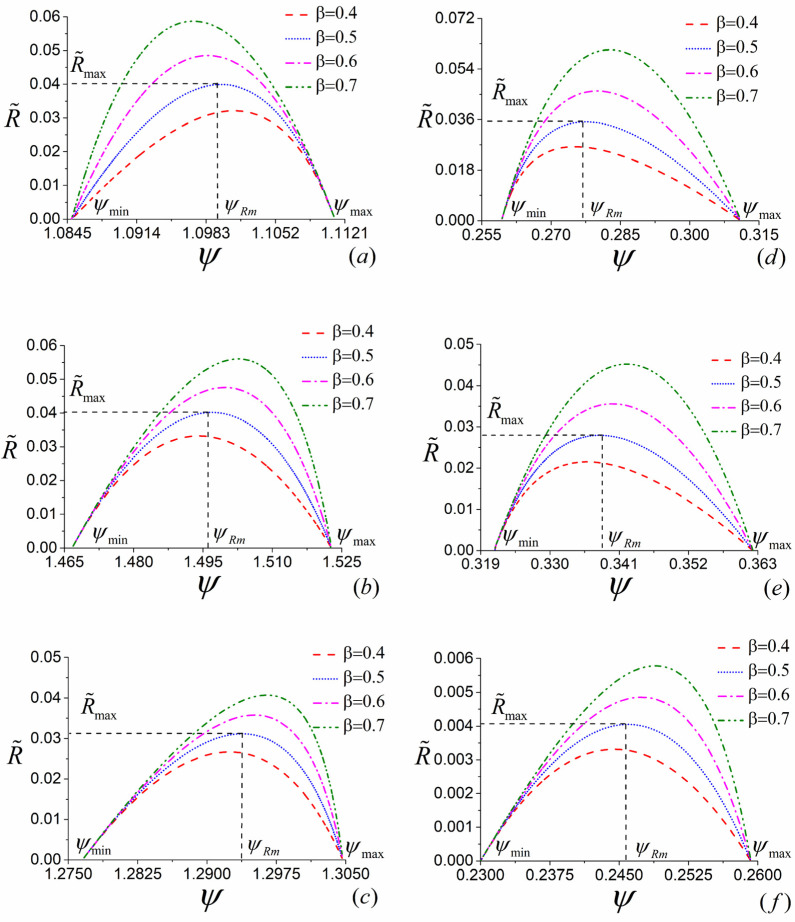
The curves of optimized ψ varying with the corresponding $\tilde{R}$ with different values of $\beta_{k,thp}$ and $\beta_{k,tht}$ (k=a,b,c) for the combined cycle models *A*, *B* and *C* of three-terminal heat pump (**a**–**c**) and heat transformer (**d**–**f**), where α=0.5 for (**a**–**e**), α=0.7 for (**f**), the other parameters have the same values as those adopted in Figure 6.

As the parameters accounting for the dissipative symmetry between two subsystems, $\beta_{k,thp}$ and $\beta_{k,tht}$ (k=a,b,c) have great influences on the performance of the three-terminal heat devices. By using Equations (5)–(7), (11)–(13), (17)–(19), (23)–(43) and the numerical calculation conducted with Mathematica, the optimal curves of the COP varying with the corresponding heating load for both the three-terminal heat pump and heat transformer with different values of β can be drawn, as shown by Figure 7a–f.

It can be found from Figure 7 that, for all different values of β, the optimal relationship between ψ and $\tilde{R}$ are not monotonic. Moreover, Figure 7 shows that the maximum and minimum values of COP, i.e., $\psi_{\max}$ and $\psi_{\min}$, are independent of the variation of β, whereas the optimal values of COP $\psi_{Rm}$ making $\tilde{R}$ attain its maximum vary with the changes of β. More specifically, $\psi_{Rm}$ increases as the corresponding value of β grows for the models *B* and *C* of the three-terminal heat pump and all three models of the three-terminal heat transformer. For model *A* of the three-terminal heat pump, $\psi_{Rm}$ decreases with the increase of β.

### 4.3. The Influence of σ

Apart from β, ${\tilde{\sigma }}_{i}$ and ${\tilde{\sigma }}_{j}$ (i=H,j=OP for (a), i=O,j=H for (b), i=HP,j=O for (c), i=H,j=OP for (d), i=HO,j=HP for (e) and i=H,j=PO for (f)) are also two important parameters denoting the dissipative symmetry inside the individual subsystems, respectively, whose effects on the performance of the overall system need to be discussed in detail.

**Figure 8:**
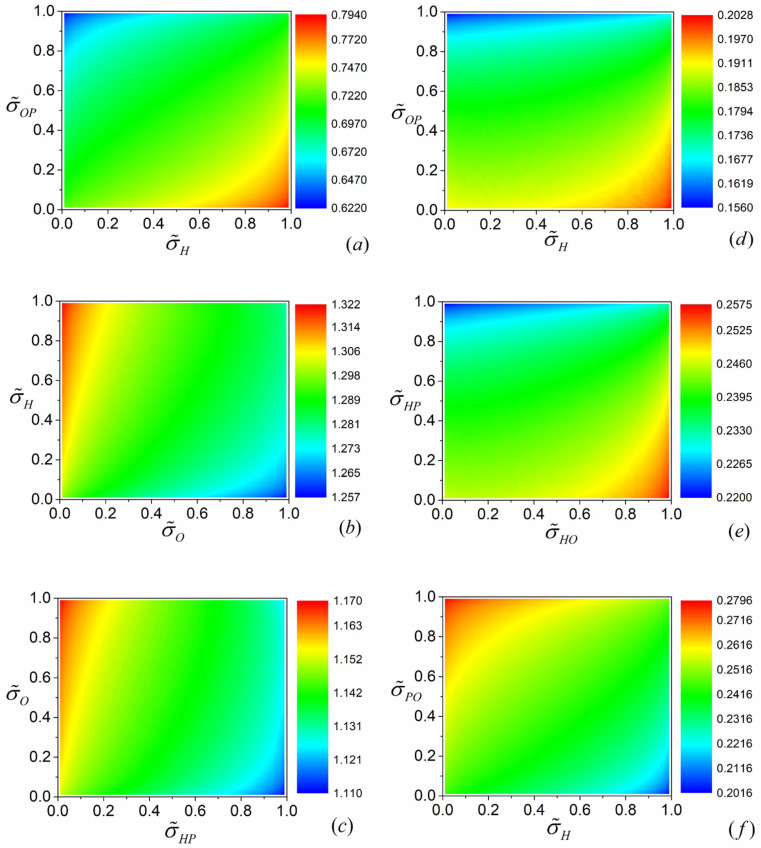
Three-dimensional projection of $\psi_{Rm}$ varying with ${\tilde{\sigma }}_{i}$ and ${\tilde{\sigma }}_{j}$ (i=H,j=OP for (**a**), i=O,j=H for (**b**), i=HP,j=O for (**c**), i=H,j=OP for (**d**), i=HO,j=HP for (**e**) and i=H,j=PO for (**f**)) for three-terminal heat pump (**a**–**c**) and three-terminal heat transformer (**d**–**f**), where $\beta_{k,thp}=\beta_{k,tht}=0.5$ (k=a,b,c); ${\tilde{T}}_{H}=1.5$, ${\tilde{T}}_{P}=1.2$ for (**a**–**c**) and ${\tilde{T}}_{H}=1.2$, ${\tilde{T}}_{P}=1.5$ for (**d**–**f**); α=0.3 for (**a**–**e**) and α=0.7 for (**f**).

By using Equations (5)–(7), (11)–(13), (17)–(19) and (23)–(43) and the numerical calculation conducted with Mathematica, the three-dimensional projections of $\psi_{Rm}$ varying with ${\tilde{\sigma }}_{i}$ and ${\tilde{\sigma }}_{j}$ for both the three-terminal heat pump and heat transformer can be plotted, which are presented by Figure 8a–f. It can be seen from Figure 8a–c that, for models *A*, *B* and *C* of the three-terminal heat pump, $\psi_{Rm}$ attains its maximum at the conditions of ${\tilde{\sigma }}_{H}\to 1$, ${\tilde{\sigma }}_{OP}\to 0$; ${\tilde{\sigma }}_{H}\to 1$, ${\tilde{\sigma }}_{O}\to 0$; and ${\tilde{\sigma }}_{HP}\to 0$, ${\tilde{\sigma }}_{O}\to 1$, respectively. In addition, the minimum values of $\psi_{Rm}$ can be reached at the conditions of ${\tilde{\sigma }}_{H}\to 0$, ${\tilde{\sigma }}_{OP}\to 1$; ${\tilde{\sigma }}_{H}\to 0$, ${\tilde{\sigma }}_{O}\to 1$; and ${\tilde{\sigma }}_{HP}\to 1$, ${\tilde{\sigma }}_{O}\to 0$. Similarly, Figure 8d–f show that the conditions of ${\tilde{\sigma }}_{H}\to 1$, ${\tilde{\sigma }}_{OP}\to 0$; ${\tilde{\sigma }}_{HO}\to 1$, ${\tilde{\sigma }}_{HP}\to 0$; and ${\tilde{\sigma }}_{H}\to 1$, ${\tilde{\sigma }}_{PO}\to 1$ correspond to the maximum values of $\psi_{Rm}$ for models *A*, *B* and *C* of the three-terminal heat transformer, respectively. In addition, the minimum points of $\psi_{Rm}$ can be approached in the limit of ${\tilde{\sigma }}_{H}\to 0$, ${\tilde{\sigma }}_{OP}\to 1$; ${\tilde{\sigma }}_{HO}\to 0$, ${\tilde{\sigma }}_{HP}\to 1$; and ${\tilde{\sigma }}_{H}\to 1$, ${\tilde{\sigma }}_{PO}\to 0$.

### 4.4. Upper and Lower Bounds

**Figure 9:**
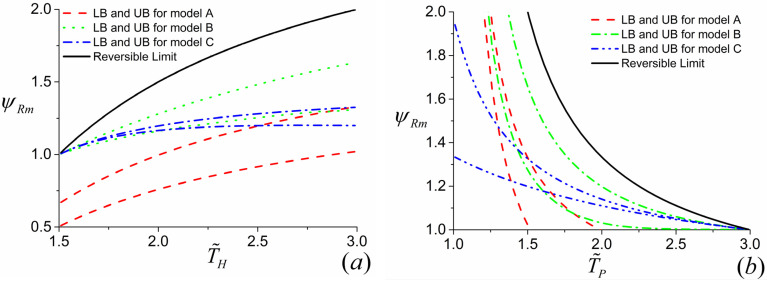
The curves of $\psi_{r}$ and $\psi_{Rm}$ varying with (**a**) ${\tilde{T}}_{H}$ and (**b**) ${\tilde{T}}_{P}$ for the three-terminal heat pump, where α=0.5, (**a**) ${\tilde{T}}_{P}=1.5$ and (**b**) ${\tilde{T}}_{H}=3$.

**Figure 10:**
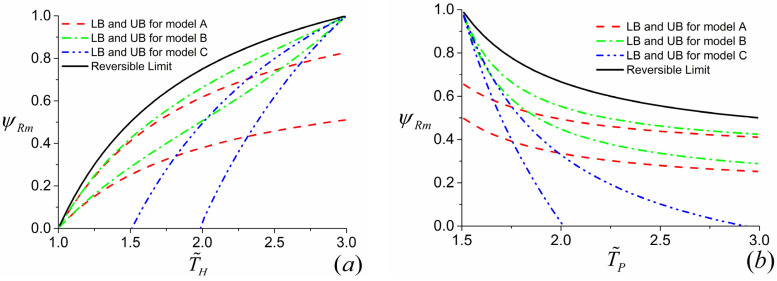
The curves of $\psi_{r}$ and $\psi_{Rm}$ varying with (**a**) ${\tilde{T}}_{H}$ and (**b**) ${\tilde{T}}_{P}$ for the three-terminal heat transformer, where α=0.5, (**a**) ${\tilde{T}}_{P}=3$ and (**b**) ${\tilde{T}}_{H}=1.5$.

By using the similar approach, the variations of $\psi_{r}$ and $\psi_{Rm}$ with ${\tilde{T}}_{H}$ and ${\tilde{T}}_{P}$ for both the three-terminal heat pump and heat transformer can be drawn. Moreover, based on the above discussions, the upper and lower bounds of $\psi_{Rm}-{\tilde{T}}_{H}$ and $\psi_{Rm}-{\tilde{T}}_{P}$ for the three-terminal heat pump can be obtained by setting $\beta_{a,thp}\to 0$, ${\tilde{\sigma }}_{H}\to 1$, ${\tilde{\sigma }}_{OP}\to 0$ (upper bound for model *A*); $\beta_{a,thp}\to 1$, ${\tilde{\sigma }}_{H}\to 0$, ${\tilde{\sigma }}_{OP}\to 1$ (lower bound for model *A*); $\beta_{b,thp}\to 1$, ${\tilde{\sigma }}_{H}\to 1$, ${\tilde{\sigma }}_{O}\to 0$ (upper bound for model *B*); $\beta_{b,thp}\to 0$, ${\tilde{\sigma }}_{H}\to 0$, ${\tilde{\sigma }}_{O}\to 1$ (lower bound for model *B*); $\beta_{c,thp}\to 1$, ${\tilde{\sigma }}_{HP}\to 0$, ${\tilde{\sigma }}_{O}\to 1$ (upper bound for model *C*); $\beta_{c,thp}\to 0$, ${\tilde{\sigma }}_{HP}\to 1$, ${\tilde{\sigma }}_{O}\to 0$ (lower bound for model *C*), respectively, which are displayed by Figure 9a,b. Likewise, the upper and lower bounds of $\psi_{Rm}-{\tilde{T}}_{H}$ and $\psi_{Rm}-{\tilde{T}}_{P}$ for the three-terminal heat transformer can be generated by setting $\beta_{a,tht}\to 1$, ${\tilde{\sigma }}_{H}\to 1$, ${\tilde{\sigma }}_{OP}\to 0$ (upper bound for model *A*); $\beta_{a,tht}\to 0$, ${\tilde{\sigma }}_{H}\to 0$, ${\tilde{\sigma }}_{OP}\to 1$ (lower bound for model *A*); $\beta_{b,tht}\to 1$, ${\tilde{\sigma }}_{HO}\to 1$, ${\tilde{\sigma }}_{HP}\to 0$ (upper bound for model *B*); $\beta_{b,tht}\to 0$, ${\tilde{\sigma }}_{HO}\to 0$, ${\tilde{\sigma }}_{HP}\to 1$ (lower bound for model *B*); $\beta_{c,tht}\to 1$, ${\tilde{\sigma }}_{H}\to 0$, ${\tilde{\sigma }}_{PO}\to 1$ (upper bound for model *C*); $\beta_{c,thp}\to 0$, ${\tilde{\sigma }}_{H}\to 1$, ${\tilde{\sigma }}_{PO}\to 0$ (lower bound for model *C*), respectively, which are depicted by Figure 10a,b.

It can be seen from Figure 9a the values of $\psi_{Rm}$ for models *A* and *B* of the three-terminal heat pump increase monotonically with the increase of for the given value of ${\tilde{T}}_{P}$, whereas for model *C*, there exists an optimal value of ${\tilde{T}}_{H}$ at which $\psi_{Rm}$ attains its maximum for the given value of ${\tilde{T}}_{P}$. It is an expected result which can be realized from Figure 2c. Figure 2c shows that, for the given value of ${\tilde{T}}_{P}$, the performance of the Carnot heat engine subsystem enhances with the increase of ${\tilde{T}}_{H}$, while the performance of the Carnot heat pump subsystem decreases as ${\tilde{T}}_{H}$ grows. Consequently, the performance of the overall system depends on the compromise between these two factors, which makes the $\psi_{Rm}-{\tilde{T}}_{H}$ curves not monotonic. In addition, Figure 9b indicates that $\psi_{Rm}$ are monotonically decreasing function of ${\tilde{T}}_{P}$ for all three models of the three-terminal heat pump with the given value of ${\tilde{T}}_{H}$.

Moreover, Figure 9a,b also show that, for models *B* and *C* of the three-terminal heat pump, the value of $\psi_{Rm}$ achieves its minimum, namely, $\psi_{Rm}=\psi_{r}=1$, at the condition of ${\tilde{T}}_{H}={\tilde{T}}_{P}$. Nevertheless, the value of $\psi_{Rm}$ for model *A* of the three-terminal heat pump can be less than 1 even at the condition of ${\tilde{T}}_{H}={\tilde{T}}_{P}$. The reasonability of the above results can be explained as follows. For models *B* and *C* of the three-terminal heat pump, when ${\tilde{T}}_{H}={\tilde{T}}_{P}$ the Carnot heat engine subsystem cannot work and the heat is directly transferred between the high-temperature reservoir and the heated space with the same temperature, which is shown by Figure 2b,c. Therefore, one has $\psi_{Rm}=\psi_{r}=1$, whereas Figure 2a shows that, for model *A* of the three-terminal heat pump, the high-temperature reservoir and the heated space are connected via two subsystems rather than one. In other words, the high-temperature reservoir and the heated space cannot be contacted directly even at the condition of ${\tilde{T}}_{H}={\tilde{T}}_{P}$. As a consequence, the irreversibilities lead to the reduction of the heat absorbed by the heated space comparing to the heat released by the high-temperature reservoir, namely, $\psi_{Rm}<1$. As pointed out above, the three-terminal heat pump with the COP less than 1 is meaningless.

As for the three-terminal heat transformer, the curves of $\psi_{Rm}-{\tilde{T}}_{H}$ are monotonically increasing and the curves of $\psi_{Rm}-{\tilde{T}}_{P}$ are monotonically decreasing for all three models, which can be seen by Figure 10a,b, respectively. It can be also found from Figure 10a,b that $\psi_{Rm}=\psi_{r}=0$ at the condition of ${\tilde{T}}_{H}=1$ (i.e., $T_{H}=T_{O}$) for models *A* and *B* and $\psi_{Rm}=\psi_{r}=1$ at the condition of ${\tilde{T}}_{H}={\tilde{T}}_{P}$ for models *B* and *C*, which is a reasonable result. For models *A* and *B* of the three-terminal heat transformer, the Carnot heat engine subsystem is disabled at the condition of ${\tilde{T}}_{H}=1$ (i.e., $T_{H}=T_{O}$) as shown by Figure 3a,b. Hence, the heat delivered to the heated space is zero ($\psi_{Rm}=\psi_{r}=0$). For models *B* and *C* of the three-terminal heat transformer, the heat is directly transferred between the high-temperature reservoir and the heated space with the same temperature at the condition of ${\tilde{T}}_{H}={\tilde{T}}_{P}$, which is shown by Figure 3b,c. Therefore, one has $\psi_{Rm}=\psi_{r}=1$. The reason why $\psi_{Rm}<1$ for model *A* of the three-terminal heat transformer at the condition of ${\tilde{T}}_{H}={\tilde{T}}_{P}$ is similar to model *A* of the three-terminal heat pump, which has been discussed in the third paragraph of the Section 4.4. In addition, Figure 10a,b also indicate that the values of $\psi_{Rm}$ for models *A* and *B* of the three-terminal heat transformer are always greater than 0. However, for model *C* of the three-terminal heat transformer, the values of $\psi_{Rm}$ can be less than 0 at some circumstances since the heated space of model *C* not only absorbs heat from the subsystem of Carnot heat pump but also releases heat into the subsystem of Carnot heat engine (Figure 3c).

More importantly, it can be seen from Figure 9 and Figure 10 that model *B* of the three-terminal heat pump and the three-terminal heat pump exhibits better performance in comparison with two other models, which is in conformity with the results obtained by Ref. [36]. In addition, the constructions of model *B* for the three-terminal heat pump and the three-terminal heat pump are accordant with the practical thermally driven heat pump [39,40,41] and thermally driven heat transformer [42,43]. More specifically, for a practical absorption heat pump, the low-grade thermal energy source with high temperature releases heat into the generator; the heated space with intermediate temperature absorbs heat from two components, namely, the condenser and absorber; the evaporator absorbs heat from environment. It can be seen from Figure 2 that the construction of model *B* of the three-terminal heat pump is the only one fitting with the above operations. Similarly, the consistency between the practical absorption heat transformer and the model *B* of the three-terminal heat transformer in Figure 3b can be also found. Consequently, the consistency between the low-dissipation model and endoreversible model for multi-terminal thermodynamic cycles is confirmed. In addition, the validity of the application of the low-dissipation model for multi-terminal devices are verified as well.

It is worth pointing out that the obtained upper and lower bounds are difficult to be validated directly by experimental and simulated works due to the lack of optimal size ratio and the difficulty in determining the values of α for practical devices. However, the rationality of the proposed low-dissipation model and the obtained results in the present paper can be realized indirectly from the following aspects. Firstly, the validity of low-dissipation thermodynamic model has been verified by many two-terminal thermal devices such as refrigerators [11], heat engines [7,12], chemical engines [15] and so on. In addition, the proposed models of the low-dissipation three-terminal heat pump systems in the present paper are composed of two low-dissipation two-terminal subsystems. More importantly, in Ref. [33], a low-dissipation thermodynamic model of three-terminal refrigerator has been established and analyzed by using the similar approach adopted in the present paper. Different from three-terminal heat pump systems, the optimal size ratio of the low-dissipation three-terminal refrigerator can be determined in Ref. [33]. Consequently, the obtained global upper and lower bounds of the COP at maximum cooling power were validated by adopting 15 sets of experimental and simulated data.

### 4.5. Extensions for the Three-Terminal Chemical Pump and Chemical Potential Transformer

The performances of the three-terminal chemical pump (potential transformer) with different constructions of a chemical pump driven by a chemical engine can be also investigated and compared within the framework of low-dissipation assumption.

More importantly, the results obtained above for the three-terminal heat pump (heat transformer) can be directly used to discuss the performances of the three-terminal chemical pump (potential transformer) by replacing the heat reservoirs (with temperatures $T_{H}$, $T_{P}$ and $T_{O}$) with chemical reservoirs (with chemical potentials $\mu_{H}$, $\mu_{P}$ and $\mu_{O}$), respectively and considering the four mass-transfer processes instead of the four heat-transfer processes. The application of low-dissipation assumption for the thermodynamic devices operating between chemical potential reservoirs have been discussed by Refs. [15,34]. Therefore, the details of the extensions will not be given for saving the length in the present paper. By using the similar approaches in the Section 3 and Section 4 above, it is not difficult to deduce that the three-terminal chemical pump and the three-terminal chemical potential transformer exhibit the best performance with the constructions like model *B* in Figure 2b and Figure 3b, respectively. It is also the consistent results with Refs. [37,38] in which endoreversible assumption is adopted to study and compare the performances of the three-terminal chemical pump (potential transformer).

## 5. Conclusions

In the present paper, the low-dissipation models of three-terminal heat pump and heat transformer with three different combined constructions have been established, respectively, which provides another approach to evaluate and compare the performances of them. The main tasks implemented and the important findings are listed as follows: 

(1) The optimal performance characteristics for various combined constructions are discussed and revealed based on the proposed low-dissipation models, respectively.

(2) The upper and lower bounds of the COP at maximum heating load for different constructions are generated and compared by introducing a parameter measuring the deviation from the reversible limit of the system.

(3) The optimal constructions for low-dissipation three-terminal heat pump and heat transformer are determined, respectively, namely, model *B* in Figure 2 and Figure 3, which are accordant with previous research and the engineering practices. Consequently, the compatibility between the low-dissipation model and endoreversible model and the validity of the application of low-dissipation model for multi-terminal thermodynamic devices are further confirmed.

It is reasonable to believe that the achievements of the present paper enrich the theoretical thermodynamic model of thermally driven heat pump systems and may provide some useful guidelines for the design and operation of realistic thermally driven heat pump and heat transformer.

## Data Availability

The data presented in this study are generated by numerical calculations based on the derived equations, which have been explicitly indicated in the paper.

## Nomenclature

Latin letters	
C	size ratio of two subsystems for the three-terminal heat pump
D	size ratio of two subsystems for the three-terminal heat transformer
Q	heat (J)
R	heating load (W)
$\tilde{R}$	dimensionless heating load
S	entropy ($JK^{-1}$)
T	temperature (K)
$\tilde{T}$	dimensionless temperature
t	time (s)
$\tilde{t}$	dimensionless time
W	work (J)
Greek letters	
α	parameter of deviation from reversible limit
β	dissipation symmetry between two subsystems
ε	coefficient of performance of Carnot heat pump subsystem
η	efficiency of Carnot heat engine subsystem
μ	chemical potential ($Jmol^{-1}$)
σ	dissipation parameter (s)
$\tilde{\sigma }$	dimensionless dissipation parameter
τ	cycle time (s)
$\tilde{\tau }$	dimensionless cycle time
ψ	coefficient of performance of the three-terminal heat pump/transformer
Subscript and Superscript	
a	model a
b	model b
c	model c
H	high temperature/chemical potential source
H, HP, HO, O, OH, OP, P, PH, PO	several heat-transfer processes in the three-terminal heat pump systems (please see Figure 2 and Figure 3)
he	Carnot heat engine subsystem
hp	Carnot heat pump subsystem
max	maximum
min	minimum
O	low temperature/chemical potential source
P	heated/pumped space
Rm	maximum heating load state
r	reversible condition
*thp*	three-terminal heat pump
tht	three-terminal heat transformer
Abbreviations	
COP	coefficient of performance
